# Alveolar Abscess

**Published:** 1862-08

**Authors:** W. H. Atkinson


ALVEOLAR ABSCESS.—W. H. Atkinson, M. D., in the *Dental Cosmos*, writes that molecular degeneracy *must* precede all idiopathic formations of pus. In traumatic lesions where a foreign body is forced within the tissues, a kind of artificial abscess is formed, an exudate partially or completely encysting it, whence, if soluble, the foreign substance is gradually absorbed, or reduced to its smallest compass. If insoluble, and located not too near centers of motion or certain important organs, it may remain inert—the abscess spontaneously cured without a rupture of the cyst. But when the molecular degeneracy is more extensive or pervading the cyst is less, or not at all defined, pus may burrow everywhere " around and beneath muscle, tendon, blood-vessels, and nerves, until the poor patient is literally one bag of illy formed pus and broken down adipose tissue, between the deep and superficial layers of fasciæ."

Cases of what is popularly called " ague in the face " are closely allied to this sort of molecular degeneracy, but recently induced by excessive fatigue, bad feeding, or deficient food, or food of poor quality, cold, or excess of indulgence in the venereal function, or depressing afflictions of the affections," and are very apt to be tedious and harrassing to patient and practitioner.

This degeneracy it is our first duty to correct by controlling the aberrant forces and restoring nutrition by better regimen and morale. General sedation of the main trunks of the vascular system, and local applications, cold, warm, or hot, to the tumefied face are recommended. These may prevent altogether, or at least localize, the formation of pus. When the abscess points, open it and dress it morning and evening for a few days with equal parts of pure creosote and tincture of iodine, gradually extending the time until the discharge resembles the white of an egg, then the treatment may be suspended, but the case should still be watched, as relapses are not unfrequent. A bit of cotton, or of cork, spunk, or soft wood, on the end of a knitting needle, will be found a convenient means of dressing the cavity—leaving the pledget in the part. If the depth is miscalculated and the cotton projects, cut it off carefully, and apply over it, between the wound and the buccal walls, another pellet of cotton, wet in tinct. arnica, diluted with four parts of water.

When it is remembered how often these so often neglected abscesses lay the foundation of caries and even general necrosis, or other diseases of the maxillæ, it will be seen that too much care can scarcely be exercised to prevent such disastrous results.—*Chicago Medical Journal.*

